# Editorial: Insights in cellular endocrinology: 2022

**DOI:** 10.3389/fendo.2023.1334003

**Published:** 2023-11-30

**Authors:** Ralf Jockers, Oreste Gualillo

**Affiliations:** ^1^ Université Paris Cité, Institut Cochin, INSERM, CNRS, Paris, France; ^2^ SERGAS (Servizo Galego de Saude) and IDIS (Instituto de Investigación Sanitaria de Santiago), The NEIRID Group (Neuroendocrine Interactions in Rheumatology and Inflammatory Diseases), Santiago University Clinical Hospital, Santiago de Compostela, Spain

**Keywords:** metabolic diseases, obesity, diabetes, gpcr, nanobodies, guanylyl cyclase C, leptin, asprosin

The importance of Cellular Endocrinology is defined by its position at the frontier of fundamental and translational science. Understanding a cellular process and its dysregulation is expected reveal the mechanisms behind endocrine diseases leading eventually to new treatment strategies. The Research Topic on the “*Insights in Cellular Endocrinology: 2022*” intends to delineate major advancements and recent developments as well as future challenges and perspectives in the field of Cellular Endocrinology. Recognized experts in the field are providing their insights in seven review articles and one original article. Major advancements are often driven by technological developments, the discovery of new players, the discovery of new functions for already known players and/or the development of new pharmacological tools and drug candidates. The various contributions of the current Research Topic cover all of these aspects.

## Contributions related to metabolic diseases

The first review article is related to metabolic diseases and focuses on the importance of the maintenance of the pancreatic insulin-producing beta-cell mass for the development of innovative therapies for diabetes. This comprehensive review by Dalle et al. gives an overview of the cellular processes involved in beta-cell death and the investigation of pharmacological molecules protecting beta-cells against dysfunction and apoptotic death. The review discusses also the challenges related to the translation of preclinical data into clinical trials including pharmacokinetic properties, toxicity, efficacy and specificity and selectivity in a physiological system.

The review article by Nakagawa and Hosoi provides an update on the well-known anorexigenic adipokine leptin by pointing out emerging research directions of this hormone. The story of leptin is a nice example on how important it is to understand the cellular basis of hormone action to understand physiological functions and elaborate therapeutic strategies.

Indeed, most obese people exhibit hyperleptinemia and are resistant to the anorexic effect of leptin, thus limiting the utility of leptin as an anti-obesity drug. As discussed by the authors, the recovery of leptin sensitivity or the bypass of leptin signaling are two main strategies of current research efforts.

The review article by Farrag et al. explores another adipokine, asprosin, discovered recently, in 2016, as a glucose sensor with central and peripheral metabolic effects. Asprosin, a member of the caudamin hormone subclass, induces hepatic glucose production and influences appetite behavior via the olfactory receptor 4M1 (OR4M1), a member of the G protein-coupled receptor (GPCR) super-family. As described in this review, a number of clinical trials investigated the correlation of circulating asprosin levels with obesity- and diabetes-related diseases and gynecologic disorders. The authors point out to the potential of asprosin as a promising candidate for both novel pharmacological treatment strategies and diagnostic tools.

The review article by Prasad et al. provides an extensive overview of the guanylyl cyclase C (GC-C)/cGMP system already known since the beginning of the 1970s for its critical role of intestinal fluid and electrolyte homeostasis in regulating microbiome composition and cross-talk with host immunity. In addition to these classical intestinal roles, the authors describe the recently discovered extraintestinal functions of GC-C signaling, such as appetite control, energy expenditure, visceral nociception, and behavioral processes.

The review article by Yepmo et al. introduces a new player in metabolic diseases, namely circular RNAs, identified in eukaryotes in 1979 in the cytoplasmic of HeLa cells. Recent studies indicate the importance of the dysregulation of circular RNAs in the onset of metabolic disorders. Prominent examples discussed by the authors are the role of circular RNAs in hepatocellular carcinoma and its role as regulators of hepatic steatosis, the main characteristic of non-alcoholic fatty liver disease.

The last contribution, an original article by Hernandez-Bustamante et al., describes the potential interplay between type 2 diabetes mellitus and tuberculosis, which both produce pulmonary anti-inflammatory glucocorticoids. These effects are counteracted by the adrenal hormone dehydroepiandrosterone (DHEA). Patients with tuberculosis have a reduced DHEA/cortisol ratio. Diabetic patients have a tripled risk of contracting tuberculosis and having inadequate response to treatment. The authors suggest that DHEA or its synthetic analogs may be potential co-adjuvant treatment in diabetes-tuberculosis comorbidities.

## Development of new tools and drug candidates

Some animals such as *Camelidae* produce single variable domains of heavy chain (VHH) antibodies, also referred to as Nanobodies (Nb). Since their discovery more than 20 years ago VHHs have attracted increasing attention with complementary applications to common antibodies. The review presented by Raynaud et al. focuses on applications of Nb in the GPCR field. Nb become very popular in this field because they recognize small and cryptic regions that are not explored by common antibodies and because of the possibility to express them as intracellular Nb. After having nicely summarized the state-of-the-art of these intracellular Nbs as new tools in the GPCR field, the authors discuss existing methodological hurdles and future therapeutic opportunities for this highly druggable class of membrane receptors.

## ‘Repurposing’ of old targets

The last contribution of this Research Topic elaborates on new function of gamma-aminobutyric acid type B (GABA_B_) receptors. These receptors belong to the GPCR super-family, are widely distributed in the brain and responds to the neurotransmitter GABA. While GABA_B_ receptors are well-known to be involved in synaptic plasticity, memory formation and nociception, Turecek et al. focus their attention in their review on the cellular distribution in the inner ear and auditory pathway of the mammalian brainstem and midbrain, which is much less studied. They advocate for an important function of GABA_B_ receptors in the auditory system and speculate on the possible involvement of the GABAergic system in various pathologies of the auditory system. Finally, they discuss the challenges of specific therapeutic intervention such as auditive loss by targeting receptors with broad expression and multiple functions as the GABA_B_ receptors.

In conclusion, we hope that this Research Topic will inspire, inform and provide direction and guidance to junior and senior researchers in the field of cellular endocrinology.

## Author contributions

RJ: Writing – original draft, Writing – review & editing. OG: Writing – original draft, Writing – review & editing.

